# Supplementary figures and images for: Anatomical Predictors of Clinical Improvement After Profundoplasty in Patients with an Occluded Superficial Artery: A Pilot Study
Source: J Clin Med. 2025 Aug 22;14(17):5938. doi: 10.3390/jcm14175938 (PMC12429361; doi:10.3390/jcm14175938)

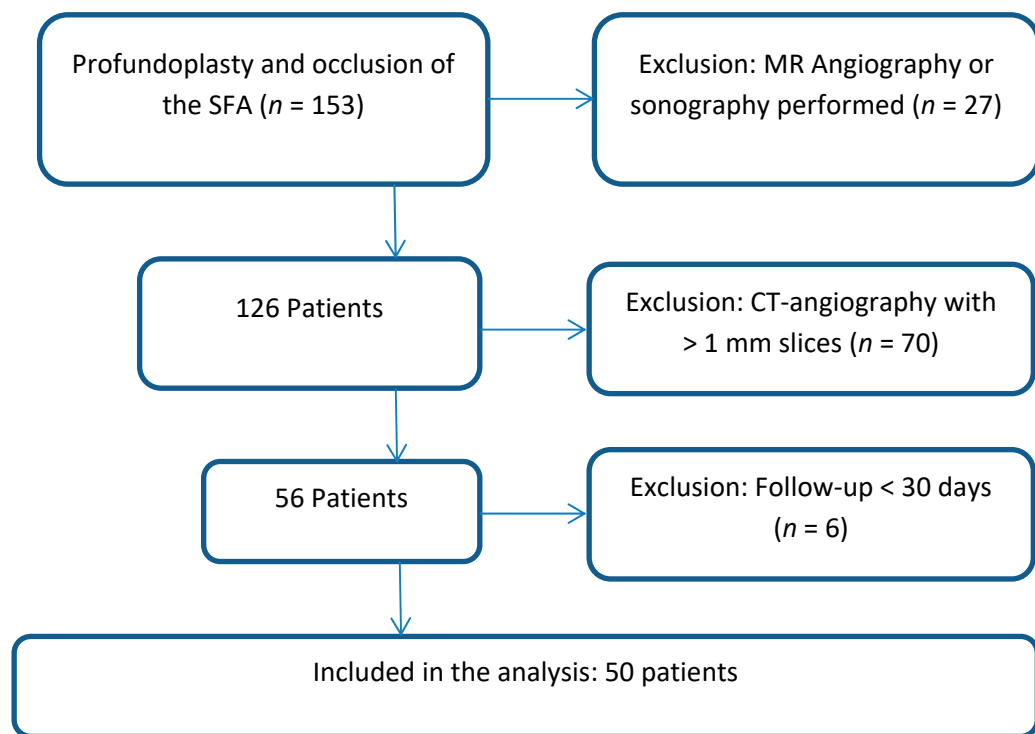

*SFA = superficial femoral artery*

Supplement: Supplementary file 1 [file jcm-14-05938-s001.zip › Figure S1.pdf]
